# Randomized double-blind phase II survival study comparing immunization with the anti-idiotypic monoclonal antibody 105AD7 against placebo in advanced colorectal cancer

**DOI:** 10.1054/bjoc.2001.1725

**Published:** 2001-06

**Authors:** C A Maxwell-Armstrong, L G Durrant, T J D Buckley, J H Scholefield, R A Robins, K Fielding, J R T Monson, P Guillou, H Calvert, J Carmichael, J D Hardcastle

**Affiliations:** Division of Gastrointestinal Surgery, University Hospital, Queens Medical Centre, Nottingham, NG7 2UH, UK; University Department of Clinical Oncology, City Hospital, Hucknall Road, Nottingham, UK; University Department of Immunology, Queens Medical Centre, Nottingham, NG7 2UH, UK; Trent Institute for Health Services Research, Queens Medical Centre, Nottingham, NG7 2UH, UK; Department of Surgery, Castle Hill Hospital, Cottingham, Hull, UK; University Department of Surgery, St James Hospital, Leeds, UK; University Department of Clinical Oncology, Newcastle General Hospital, Newcastle, UK

**Keywords:** anti-idiotypic antibody, colorectal cancer, immunotherapy, 105AD7

## Abstract

The cancer vaccine 105AD7 is an anti-idiotypic monoclonal antibody that mimics the tumour-associated antigen 791T/gp72 (CD55, Decay Accelerating Factor) on colorectal cancer cells. Phase I studies in patients with advanced disease confirmed that 105AD7 is non-toxic, and that T cell responses could be generated. A prospective, randomized, double-blind, placebo-controlled survival study in patients with advanced colorectal cancer was performed. 162 patients were enrolled between April 1994 and October 1996. Patients attended at trial entry, and at 6 and 12 weeks, where they received 105AD7 or placebo. Study groups were comparable in terms of patient demographics, and time from diagnosis of advanced colorectal cancer (277.1 v 278.6 days). Baseline disease was similar, with 50% of patients having malignancy in at least 2 anatomic sites. Compliance with treatment was poor, with only 50% of patients receiving 3 planned vaccinations. Median survival from randomization date was 124 and 184 days in 105AD7 and placebo arms respectively (*P* = 0.38), and 456 and 486 days from the date of diagnosis of advanced disease (*P* = 0.82). 105AD7 vaccination does not prolong survival in patients with advanced colorectal cancer. The reasons for lack of efficacy are unclear, but may reflect the high tumour burden in the patient population, and poor compliance with immunization. Further vaccine studies should concentrate on patients with minimal residual disease. © 2001 Cancer Research Campaign http://www.bjcancer.com


British Journal of Cancer (2001) 84(11), 1443–1446
© 2001 Cancer Research Campaign
doi: 10.1054/ bjoc.2001.1725, available online at http://www.idealibrary.com on IDEAL®

http://www.bjcancer.com

### Randomized double-blind phase II survival study comparing immunization with the anti-idiotypic monoclonal antibody 105AD7 against placebo in advanced colorectal cancer

**CA Maxwell-Armstrong[1], LG Durrant[2], TJD Buckley[1], JH Scholefield[1], RA Robins[3], K Fielding[4], JRT Monson[5], P Guillou[6], H Calvert[7], J Carmichael[2] and JD Hardcastle[1]**

[1]Division of Gastrointestinal Surgery, University Hospital, Queens Medical Centre, Nottingham, NG7 2UH, UK; [2]University Department of Clinical Oncology, City Hospital, Hucknall Road, Nottingham, UK; [3]University Department of Immunology, Queens Medical Centre, Nottingham, NG7 2UH, UK; [4]Trent Institute for Health Services Research, Queens Medical Centre, Nottingham, NG7 2UH, UK, [5]Department of Surgery, Castle Hill Hospital, Cottingham, Hull, UK; [6]University Department of Surgery, St James Hospital, Leeds, UK; [7]University Department of Clinical Oncology, Newcastle General Hospital, Newcastle, UK

**Summary** The cancer vaccine 105AD7 is an anti-idiotypic monoclonal antibody that mimics the tumour-associated antigen 791T/gp72 (CD55, Decay Accelerating Factor) on colorectal cancer cells. Phase I studies in patients with advanced disease confirmed that 105AD7 is non-toxic, and that T cell responses could be generated. A prospective, randomized, double-blind, placebo-controlled survival study in patients with advanced colorectal cancer was performed. 162 patients were enrolled between April 1994 and October 1996. Patients attended at trial entry, and at 6 and 12 weeks, where they received 105AD7 or placebo. Study groups were comparable in terms of patient demographics, and time from diagnosis of advanced colorectal cancer (277.1 v 278.6 days). Baseline disease was similar, with 50% of patients having malignancy in at least 2 anatomic sites. Compliance with treatment was poor, with only 50% of patients receiving 3 planned vaccinations. Median survival from randomization date was 124 and 184 days in 105AD7 and placebo arms respectively ($P = 0.38$), and 456 and 486 days from the date of diagnosis of advanced disease ($P = 0.82$). 105AD7 vaccination does not prolong survival in patients with advanced colorectal cancer. The reasons for lack of efficacy are unclear, but may reflect the high tumour burden in the patient population, and poor compliance with immunization. Further vaccine studies should concentrate on patients with minimal residual disease. © 2001 Cancer Research Campaign http://www.bjcancer.com

**Keywords**: anti-idiotypic antibody; colorectal cancer; immunotherapy; 105AD7

Anti-idiotypic antibodies that bind at the active site of an anti-tumour antibody have the potential to act as surrogate antigens, thus stimulating immune responses against tumour antigens. 105AD7 is a human monoclonal anti-idiotypic antibody that was derived from a cancer patient (Austin et al, 1989) who had received radiolabelled 791T/36 antibody for diagnostic imaging of his colorectal cancer metastases. The 791T/36 antibody has recently been shown to bind to CD55 (Spendlove et al, 1999) a complement regulatory protein. This antigen is over-expressed by tumours presumably to protect them from complement-mediated lysis. 105AD7 has been shown to bind at the combining site of 791T/36 and can thus mimic CD55.

A phase I clinical trial with 105AD7 precipitated on alum showed that anti-tumour T cell responses could be induced in advanced colorectal cancer patients with no associated toxicity. T cell responses included antigen-specific blastogenesis, enhanced serum IL-2 production (Robins et al, 1991) and activation of both helper and cytotoxic T cells (Durrant et al, 1999). Furthermore survival of these patients was impressive (12 months) in a patient population with extensive liver metastases. This was significantly longer than contemporary control patients (Denton et al, 1994). A second trial evaluated 105AD7 in the neoadjuvant setting. Patients with primary colorectal cancer were immunized at diagnosis and then boosted post surgical resection. Results showed significant infiltration of helper T cells and natural killer cells (Durrant et al, 2000a, 2000b) expressing the activation marker CD25 (Maxwell-Armstrong et al, 1999) at the tumour site relative to controls. This was associated with an increase in tumour cell apoptosis in trial patients (Amin et al, 2000). Furthermore enhanced tumour killing by both natural killer cells and T cells has been seen following immunization with 105AD7 (Durrant et al, 1994). The apparent survival benefit observed in the Phase I study was therefore evaluated in a double blind randomized trial of 105AD7 antibody precipitated on alum versus an alum control vaccination.

*Received 16 August 2000*
*Revised 14 December 2000*
*Accepted 22 January 2001*

*Correspondence to*: CA Maxwell-Armstrong

### MATERIALS AND METHODS

A randomized, double blind, placebo-controlled study was performed comparing 105AD7 on alum treatment against alum placebo alone.

### Patients

Entry criteria included: patients with colorectal carcinoma with evidence of a histologically confirmed inoperable recurrence, or the presence of multiple metastatic lesions on imaging; a minimum life expectancy of 3 months, and a World Health Organization (WHO) performance grading of 0–2. Exclusion criteria included acute intercurrent illness and autoimmune or haematological disorders. Patients had not received chemotherapy in the previous 3 months, or radiotherapy within 1 month of commencement of the trial. Written informed consent was obtained from all patients, and the trial approved by local ethics committees.

### Clinical protocol

Patients were recruited in 1 of 4 participating centres (Nottingham, Leeds, Newcastle and Hull), between April 1994 and October 1996. At trial entry patient demographic data was collected, baseline disease assessed, and WHO performance status recorded. Patients received 10 μg of 105AD7/placebo intradermally and 100 μg of 105AD7/placebo intramuscularly at trial entry, 6 and 12 weeks. All vaccinations were given into the shoulder. Routine blood tests were performed prior to immunization (full blood count, urea and electrolytes, liver function tests and carcinoembryonic antigen). At trial entry and 12 weeks, a chest X-ray and CT scan were performed where possible. On each visit weight and performance status were recorded, and any toxicity or adverse events related to use of the 105AD7/placebo noted. Any new medication or treatment instituted while on study was recorded. Patients were not routinely followed up following the third dose of 105AD7/placebo.

### Monoclonal antibody

Clinical grade monoclonal antibody was prepared as previously described (Robins et al, 1991), using the guidelines of the Cancer Research Campaign. 100 μg and 10 μg vials of 105AD7 were indistinguishable to the naked eye from alum placebo. Stratified randomization was in blocks of 6, by centre.

**Table 1** Demographics of patients recruited

| | | 105AD7 *n* = 85 | Placebo *n* = 77 |
|---|---|---|---|
| Age | Mean (SD) | 63.3 (11.9) | 62.2 (11.2) |
| | Range | 27–85 | 33–85 |
| Sex | Male | 51 | 40 |
| | Female | 34 | 37 |
| Grade of primary tumour | Well | 3 | 4 |
| | Moderate/well | 3 | 2 |
| | Moderate | 43 | 42 |
| | Moderate/poor | 1 | 3 |
| | Poor | 5 | 8 |
| | Missing | 30 | 18 |
| Dukes stage of primary tumour | A | 2 | 2 |
| | B | 14 | 13 |
| | C | 35 | 33 |
| | D | 26 | 26 |
| | Missing | 8 | 3 |
| Site of primary tumour | Colon | 48 | 46 |
| | Rectum | 36 | 31 |
| | Missing | 1 | 0 |

### Statistical analysis

It was calculated that to demonstrate a 22% improvement in survival at the level $P < 0.05$, and a power of 90%, 161 patients would need to be randomized into the 2 arms of the study. This formed the basis of our sample size.

SAS (version 6.0) was used for all data summary and analysis. Survival in days was calculated from the date of randomization to date of death or censoring, whichever came first. The censoring date was taken as 21 February 1997. A secondary analysis was also carried out where survival was measured from date of diagnosis of advanced disease to date of death or censoring. Survival curves were constructed using the Kaplan–Meier method and compared using the log rank test, and a Cox proportional hazards method was used in the multivariate analysis. All $P$ values were two-sided; $P$ values $\leq 0.05$ were considered statistically significant.

### RESULTS

A total of 162 patients were enrolled in the 4 trial centres, between April 1994 and October 1996. 85 patients received 105AD7, and 77 alum placebo. 105AD7 and placebo arms were comparable in terms of age, sex ratio, and grade, stage and site of tumour (Table 1). The mean time from diagnosis of advanced disease, and date of randomization was almost identical in the 2 groups – 277.1 and 278.6 days, respectively. Time from resection of primary tumour to date of randomization was 640.8 days in patients receiving 105AD7, and 803.7 days in placebo. Baseline disease was similar in both groups, with the majority of patients having liver or lung metastases (Table 2). Disease was not confined to 1 anatomic site, and approximately 50% of patients had disease in 2 or more distinct anatomic areas (Table 3). Overall compliance was poor, with early progression resulting in only 54% and 60% of patients receiving 3 doses of 105AD7/placebo respectively (Table 4). Adjuvant radiotherapy was given to 10 patients in both 105AD7 and placebo arms, and in 13 patients in each group, as treatment for advanced disease. 11 patients immunized with 105AD7 received adjuvant chemotherapy, as compared with 13 in placebo patients. 30 and 21 patients in 105AD7 and placebo groups received chemotherapy as treatment for their advanced disease.

**Table 2** Baseline disease at date of randomization

| | 105AD7 | Placebo |
|---|---|---|
| Liver metastases | 61 (72) | 50 (65) |
| Lung metastases | 27 (32) | 20 (26) |
| Primary tumour | 10 (12) | 6 (8) |
| Local recurrence | 19 (22) | 30 (39) |
| Regional nodes | 13 (15) | 10 (13) |
| Bone metastases | 5 (6) | 1 (1) |
| Skin metastases | 2 (2) | 2 (3) |
| Brain metastases | 1 (1) | 1 (1) |
| Malignant ascites | 3 (4) | 3 (4) |
| Soft tissue metastases | 0 | 4 (5) |
| Peritoneal metastases | 9 (11) | 7 (9) |
| Other | 2 (2) | 3 (4) |

Figures in parentheses represent percentages.

© 2001 Cancer Research Campaign

**Table 3** Number of anatomical sites involved by tumour at date of randomization

| Number of anatomical sites involved | 105AD7 | Placebo |
|---|---|---|
| 1 | 41 (48) | 36 (47) |
| 2 | 26 (31) | 26 (34) |
| 3 | 10 (12) | 7 (9) |
| 4 | 2 (2) | 5 (6) |
| 5 | 4 (5) | 3 (4) |
| 6 | 2 (2) | 0 |

Figures in parentheses represent percentages.

**Table 4** Overall compliance in patients recruited

| | 105AD7 | Placebo |
|---|---|---|
| Trial entry | 85 (100) | 77 (100) |
| Week 6 | 63 (74) | 59 (77) |
| Week 12 | 46 (54) | 46 (60) |

Figures in parentheses represent percentages.

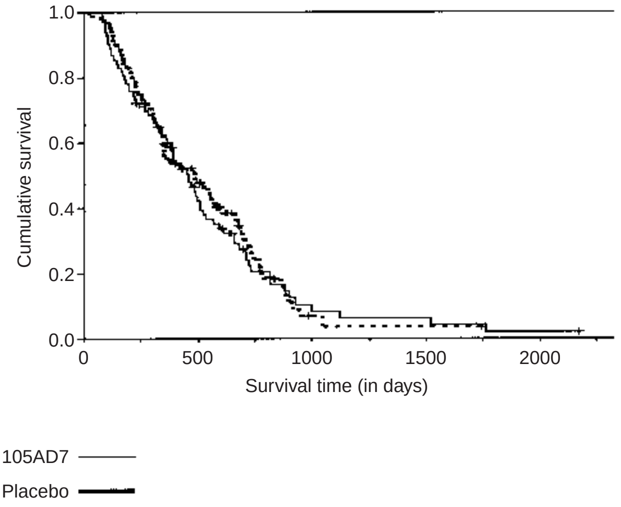

**Figure 2** Survival functions by treatment group, based on time from diagnosis of advanced disease

The number of deaths from the date of randomization was 71 and 63 in 105AD7 and placebo arms respectively. There were 3 serious adverse events (SAE) – 2 in the placebo, and 1 in the 105AD7 arm. These were classified as CTC grade 3, and all were felt unlikely to be due to 105AD7. As might be expected from a group of patients with advanced disease, WHO performance status gradually deteriorated throughout the study period.

The median survival from the date of randomization in the univariate analysis was 124 and 184 days in the 105AD7 and placebo groups respectively ($P$ = 0.38), as compared with figures of 456 and 486 days from the date of diagnosis of advanced disease ($P$ = 0.82) (Figures 1 and 2). A similar analysis was performed restricted to patients who had received 2 or more doses of vaccine/placebo. Median survivals from randomization date were 213 and 239 days in 105AD7 and control groups ($P$ = 0.69), and from diagnosis of advanced disease 511 and 486 days respectively ($P$ = 0.60).

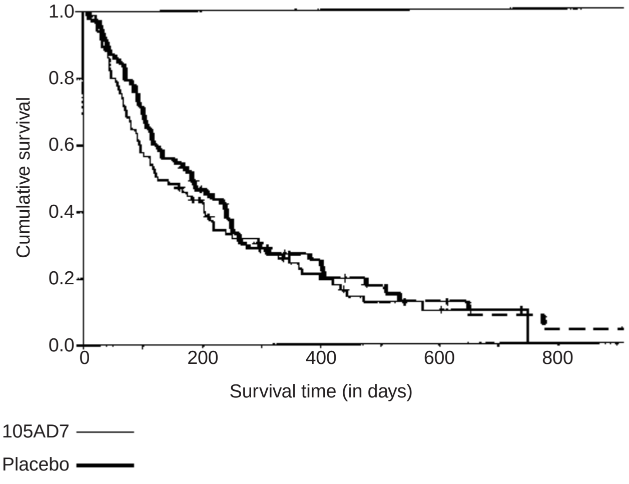

**Figure 1** Survival functions by treatment group, based on time from entry into study

A multivariate analysis was performed both on an intention to treat basis, and restricted to patients who had received at least 2 doses. These were performed from trial entry, and date of diagnosis of advanced disease. The only variables found to significantly prolong survival were chemotherapy and radiotherapy, as would be expected, and the absence of liver metastases. Mean values for urea and electrolytes remained within normal limits in both trial and control patients throughout the duration of the study. Mean haemoglobin scores were low, confirming that patients were anaemic when recruited, and that lymphocyte levels were at the lower end of the normal range.

### DISCUSSION

This study shows that immunization with the anti-idiotypic monoclonal antibody 105AD7 was well tolerated but did not confer a survival advantage on patients with advanced colorectal cancer. Patient characteristics were largely comparable in the 2 groups in terms of age, sex, site and Duke’s stage of primary tumour. Median times from diagnosis of advanced disease and inclusion into the study were similar (172 and 179 days in 105AD7 and placebo arms respectively). Interestingly the time between resection of the primary tumour and trial entry was almost twice as long in the placebo arm (13.7 months v 22.8 months). None of the patients in the Phase I study received chemotherapy and thus survival from diagnosis of advanced disease to death was very short. In contrast, approximately 30% of patients in this trial received chemotherapy as treatment for their advanced disease, and a multivariate analysis showed that this significantly improved survival. The overall time from date of diagnosis of advanced disease to death in patients in this study was now 17 months, suggesting that 105AD7 immunization was not commenced until very late in the disease course. Only half of the patients survived long enough to receive the 3 planned doses of vaccine at 0, 6 and 12 weeks. In contrast, patients in the phase I study could receive an immunization every 6 weeks until death, with most patients receiving between 3 and 7 vaccinations. Our recent studies have shown that with one exception patients do not appear to make a memory response to 105AD7 but

© 2001 Cancer Research Campaign show a brisk immune response following each immunization (Durrant et al 2000a) Thus the late stage of the colorectal cancer disease and the low number of injections may have contributed to the lack of survival benefit of 105AD7 in this trial. In addition over 50% of patients had significant tumour burdens, with disease in between 2 and 6 distinct sites. It has been hypothesized that as disease advances the efficacy of immunotherapeutic agents decreases (Jacob et al, 1997).

Since the inception of this study we have immunized over 70 potentially less immunosuppressed patients with primary colorectal cancer, and found evidence of a number of immune responses. These include autologous tumour cell killing (Durrant et al, 1994), upregulation of the IL-2 receptor (Buckley et al, 1995; Maxwell-Armstrong et al, 1999) and significant infiltration of CD4+ lymphocytes and NK cells (Durrant et al, 2000b). These responses have correlated with increased apoptosis of tumour cells in trial patients relative to controls (Amin et al, 2000). These findings are of interest, and suggest an immune response may be developing in patients with minimal residual disease. This is one area where future activity will concentrate, though clearly the bottom line is whether patients live longer or not.

This trial highlights the problem of assessing the efficacy of cancer vaccines in advanced disease patients – clearly there was no evidence of survival advantage in this randomized study. It may be difficult to generate a sustained immune response such that the balance between activated T cells and tumour burden is tipped in favour of tumour regression. Furthermore recent evidence suggests that if naive T cells receive a signal from MHC/peptide without a constimulatory signal they can be anergized. As tumours express MHC/peptide in the absence of costimulation they will effectively anergize naive T cells reducing the pool of T cells available for stimulation by a vaccine. More aggressive immunization with a stronger Th1 adjuvant than alum may help rectify the balance. Future studies are therefore using BCG as an adjuvant and reconfiguring the anti-idiotype as a DNA vaccine. It remains to be seen if these approaches can be shown to be of benefit, though clearly persevering with our current vaccine in patients with advanced disease would not be sensible.

The apparent survival advantage of immunization with 105AD7 observed in our initial phase I trial was not confirmed in a double blind randomized trial. However 105AD7 does induce significant tumour cell apoptosis in a neoadjuvant trial. More aggressive immunization with a more potent immune adjuvant in the treatment of patients with minimal residual disease is therefore planned.

### ACKNOWLEDGEMENTS

The authors would like to acknowledge the support of the clinicians throughout the UK who referred patients for inclusion in this study. This work was funded by the Cancer Research Campaign, 10 Cambridge Terrace, Regents Park, London.

### REFERENCES

Amin S, Robins RA, Maxwell-Armstrong CA, Galvin A, Scholefield JH and Durrant LG (2000) Vaccine induced apoptosis – A novel clinical trial end point? *Cancer Res* **60**: 3132–3136

Austin EB, Robins RA, Durrant LG, et al (1989) Human monoclonal anti-idiotypic antibody to the tumour-associated antibody 791T/36. *Immunology* **67**: 525–530

Buckley TJD, Robins RA and Durrant LG (1995) Clinical evidence that the human monoclonal anti-idiotypic antibody 105AD7 delays tumour growth by stimulating anti-tumor T cell responses. *Hum Antibod Hybridomas* **6**: 68–72

Denton GWL, Durrant LG, Hardcastle JD, et al (1994) Clinical outcome of colorectal cancer patients treated with human monoclonal anti-idiotypic antibody. *Int J Cancer* **57**: 10–14

Durrant LG, Buckley TJD, Denton GWL, et al: (1994) Enhanced cell-mediated killing in patients immunized with a human monoclonal anti-idiotypic antibody 105AD7. *Cancer Res* **54**: 4837–4840

Durrant LG, Buckley DJ, Robins RA et al (2000a) 105AD7 cancer vaccine stimulates anti-tumour helper and cytotoxic T cell responses in colorectal cancer patients but repeated immunisations are required to maintain these responses. *Int J Cancer* **85**: 87–92

Durrant LG, Maxwell-Armstrong CA, Buckley D, et al (2000b) A neoadjuvant clinical trial in colorectal cancer patients of the human anti-idiotypic antibody 105AD7, that mimics CD55. *Clinical Cancer Research* **6**: 422–430

Jacob L, Somasundaram R, Smith W, et al (1997) Cytotoxic T cell clone against rectal carcinoma induced by stimulation of a patients peripheral blood mononuclear cells with autologous cultured tumour cells. *Int J Cancer* **71**: 325–332

Maxwell-Armstrong CA, Durrant LG, et al Robins RA, et al (1999) Increased activation of lymphocytes infiltrating primary colorectal cancers following immunisation with the anti-idiotypic monoclonal antibody 105AD7. *Gut* **45**: 593–598

Robins RA, Denton GWL, Hardcastle JD, et al (1991) Antitumor immune response and Interleukin-2 production induced in colorectal cancer patienta by immunisation with human monoclonal anti-idiotypic antibody. *Cancer Res* **51**: 5425–5429

Spendlove I, Li L, Carmichael J, et al (1999) Decay Accelerating Factor (CD55): A Target for Cancer Vaccines. *Cancer Res* **59**: 2282–2286

© 2001 Cancer Research Campaign
